# Predictors of Complete Response in Patients with Hepatocellular Carcinoma Treated with Trans-Arterial Radioembolization

**DOI:** 10.3390/curroncol28010095

**Published:** 2021-02-21

**Authors:** Yuna Kim, Jae Seung Lee, Hye Won Lee, Beom Kyung Kim, Jun Yong Park, Do Young Kim, Sang Hoon Ahn, Seung Up Kim

**Affiliations:** 1 Department of Internal Medicine, Yonsei University College of Medicine, Seoul 03722, Korea; sadts@yuhs.ac (Y.K.); sikarue@yuhs.ac (J.S.L.); lorry-lee@yuhs.ac (H.W.L.); beomkkim@yuhs.ac (B.K.K.); drpjy@yuhs.ac (J.Y.P.); dyk1025@yuhs.ac (D.Y.K.); AHNSH@yush.ac (S.H.A.); 2 Yonsei Liver Center, Severance Hospital, Seoul 03722, Korea; 3 Institute of Gastroenterology, Yonsei University College of Medicine, Seoul 03722, Korea

**Keywords:** hepatocellular carcinoma, trans-arterial radioembolization, radioembolization, complete response, outcome, predictor

## Abstract

Background: Trans-arterial radioembolization (TARE) has shown promising results in treating hepatocellular carcinoma (HCC). We identified independent predictors of radiological complete response (CR) in patients with intrahepatic HCC who were treated with TARE. Methods: Patients with intrahepatic HCC treated with TARE between 2011 and 2017 were recruited. CR was defined according to the modified Response Evaluation Criteria in Solid Tumors. Cox regression analysis was used to determine independent predictors of CR. Results: The median age of study participants (83 men and 19 women) was 64.3 years. The mean survival after TARE was 55.5 months, and 21 (20.6%) patients died during the study period. Patients who achieved CR (14 patients, 13.7%) had significantly higher serum albumin level (median 4.1 vs. 3.9 g/dL), lower total bilirubin level (median 0.6 vs. 0.7 mg/dL), lower aspartate aminotransferase level (median 30.0 vs. 43.0 IU/L), lower alkaline phosphatase level (median 79.0 vs. 103.0 IU/L), lower alpha-fetoprotein level (median 12.7 vs. 39.9 ng/mL), lower des-gamma-carboxyprothrombin level (median 575.5 vs. 2772.0 mAU/mL), lower model for end-stage liver disease (MELD) score (median 6.0 vs. 7.0), and smaller maximal tumor diameter (median 6.3 vs. 9.0 cm) compared to those who did not achieve CR (all *p* < 0.005). Multivariate Cox regression analysis showed that lower MELD score (hazard ratio (HR) = 0.436, *p* = 0.015) and maximal tumor size < 9 cm (HR = 11.180, *p* = 0.020) were independent predictors of an increased probability of radiological CR after TARE. Conclusions: Low MELD score and small maximal tumor size were independently associated with an increased probability of CR after TARE in patients with intrahepatic HCC.

## 1. Introduction

Hepatocellular carcinoma (HCC) is one of the well-known leading causes of cancer-related deaths worldwide [1]. Despite the improvements in surveillance protocols, which have increased the diagnosis of early HCC [2,3], a considerable proportion of patients with intrahepatic HCC are not considered candidates for curative treatments such as local ablation, surgical resection, and liver transplantation, for reasons such as decreased liver function, large tumor burden, and vascular invasion [4,5,6].

Although conventional trans-arterial chemoembolization (TACE) is widely used for the treatment of HCC in the intermediate and advanced stages, the procedure has limited application due to the risk of procedure-related liver failure and complications, especially in patients of old age with large tumor or portal vein invasion [7]. Therefore, trans-arterial radioembolization (TARE), which involves intra-arterial injection of radioactive substances such as yittrium-90 (^90^Y)-loaded microspheres and minimally modifies hepatic arterial flow (compared to TACE), can be a safe alternative treatment for patients with post-TACE risks [8,9,10]. In addition, several previous studies have shown favorable outcomes, including significantly longer time to progression, better local tumor control, and higher quality of life, after TARE than after TACE [10,11,12,13,14].

TARE generally shows a delayed response in imaging studies, with confounding from true responses (e.g., post-treatment inflammation, transient increase in lesion size associated with successful necrosis, peritumoral changes, and hemorrhage in the necrotic tumor) in some cases [15,16,17,18]. Although favorable response based on arterial enhancement criteria, such as the modified Response Evaluation Criteria in Solid Tumors (mRECIST), predicts good prognostic outcomes for several HCC therapies [19,20,21,22,23], whether such enhancement criteria are suitable for the response evaluation of HCC treated with TARE has not been fully validated. Moreover, recent studies have shown that achieving complete response (CR) after TARE based on the enhancement criteria can mean a real complete cure; and even if does not, TARE can serve as a bridging therapy for other curative options [17,24]. However, the predictors of favorable response in patients treated with TARE still remain not well-known.

In this study, we aimed to identify the independent predictors of mRECIST-based CR in patients with intrahepatic HCC treated with TARE, in order to identify the optimal candidates who would benefit from TARE.

## 2. Methods

### 2.1. Patients

We retrospectively reviewed the medical records of patients with HCC who were treated with TARE at Severance Hospital, Yonsei University College of Medicine between 2011 and 2017.

Exclusion criteria were as follows: (1) age < 19 years; (2) Eastern Cooperative Oncology Group performance status score > 2; (3) presence of extrahepatic HCC; (4) presence of ascites; (5) significant extrahepatic disease representing an imminent life-threatening outcome; (6) uncontrolled medical comorbidities; (7) mortality of unknown cause that was not due to illness; (8) mortality that was absolutely due to procedure-related complications; (9) follow-up loss or death within 3 months after TARE treatment; (10) other treatment modalities such as TACE, intra-arterial chemotherapy, and surgical resection within 6 months after TARE treatment, or excessively high Lipiodol uptake due to previous TACE treatment that confounded response evaluation.

The study protocol was in accordance with the 1975 Declaration of Helsinki guidelines. Due to the retrospective nature of this study, the need for written informed consent was waived. The study protocol was approved by the Institutional Review Board and Hospital Research Ethics Committee of Severance Hospital.

### 2.2. Diagnosis and Staging

HCC was diagnosed histo-pathologically or clinically, according to the guidelines proposed by the Korea Liver Cancer Study group. The typical imaging hallmarks of HCC on dynamic computed tomography (CT) or magnetic resonance imaging (MRI) were described as arterial phase hyper-enhancement with washout in the portal venous, delayed, or hepatobiliary phases [25].

### 2.3. Yttrium-90 Radioembolization

A pre-delivery angiographic mapping procedure (i.e., angiography with technetium-99 macro-aggregated albumin scanning) was performed to evaluate the hepatic artery, enteric collaterals within the anticipated arterial treatment zone, and hepatopulmonary shunt. TARE was performed with resin (SIR-Spheres^®^; Sirtex Medical, Sydney, Australia) or glass particles (TheraSphere^®^; Biocompatibles UK Ltd., Surrey, UK) loaded with ^90^Y. The dose was determined based on the planning angiogram and prepared in the nuclear medicine department, according to the preparation guideline provided by the manufacturer. The target absorbed radiation doses to the normal liver and lungs should not exceed 70 and 25 Gy, respectively. The entire process of TARE was conducted according to previous guidelines [26].

### 2.4. Assessment of Treatment Responses Using mRECIST

The treatment responses were assessed at 1,3, and 5–6 months after TARE sessions, using liver dynamic CT or MRI with physical examination and blood tests. Thereafter, treatment responses were further assessed at intervals of 2–3 months. Two independent radiologists analyzed the images to minimize the possibility of false categorization.

Modified RECIST was used for response evaluation. For target lesions, tumor response was defined as a CR, indicated by the complete disappearance of viable lesions, or a radiological partial response (PR), defined as a 30% decrease from baseline. Radiological progressive disease (PD) was defined as a 20% increase from baseline or the presence of newly developed lesions. Radiological stable disease (SD) was defined as an increase/decrease of the percentage between PD and PR [19]. In addition to CR, PR, SD, and PR, the “best response” was defined as the most favorable response during 6 months after TARE.

### 2.5. Statistical Analysis

Patient characteristics were summarized by mean ± SD, median and range of continuous variables, and percentages for categorical variables. To compare quantitative variables, Student’s t-test or Mann-Whitney test was used. Chi-square test or Fisher’s exact test was used to compare qualitative variables. ROC analyses were used to dichotomize several continuous variables. For example, we used the rounded cutoff value 9 cm for tumor size based on the calculated cutoff value of 9.1 cm (sensitivity 52.4% and specificity 67.9%), and MELD score of 6 points based on the calculated cutoff value of 6.5 points (sensitivity 85.7% and specificity 34.6%), which maximized the sum of sensitivity and specificity.

Overall survival (OS) was estimated using Kaplan-Meier analysis. OS was defined as the time interval between the date of TARE and death, or to the last follow-up date for patients who were still alive at the time of analysis. Survival curves were compared using the log-rank test. The event of interest was CR: univariate analysis was performed for all of the variables in this study, and then multivariable analysis using the Cox proportional hazards model was used to explore the independent predictors.

A *p* < 0.05 was considered statistically significant, with a confidence interval (CI) of 95%. All statistical analyses were performed using the IBM SPSS Statistics software package version 23.0.0.0. (IBM SPSS Inc., Chicago, IL, USA).

## 3. Results

### 3.1. Patient Characteristics

**Figure 1:**
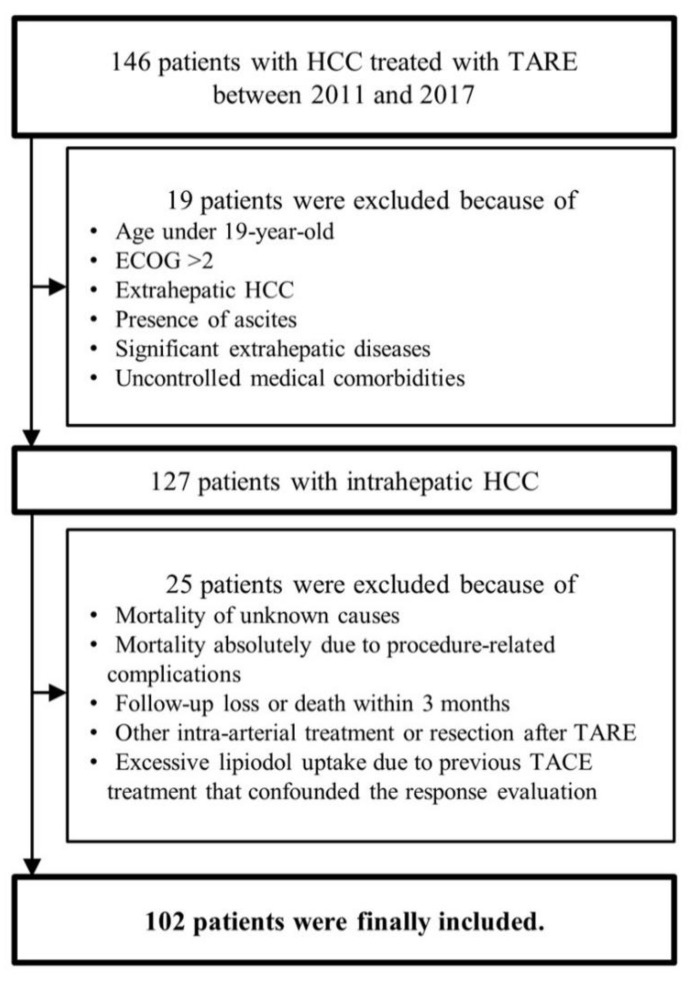
Flow of the study population selection. HCC, hepatocellular carcinoma; TARE, trans-arterial radioembolization; TACE, trans-arterial chemoembolization; ECOG, Eastern Cooperative Oncology Group.

The flow of study population selection is described in Figure 1. A total of 146 patients treated with TARE for intrahepatic HCC were recruited. After excluding 44 patients who met our exclusion criteria, 102 patients were finally selected for our study.

The baseline characteristics of the study population (83 (81.4%) men and 19 (18.6%) women) at the time of TARE are shown in Table 1. The median patient age was 64.3 years. Sixty-one (55.5%) patients had liver cirrhosis and 71 (69.6%) patients had viral hepatitis. All patients had an Eastern Cooperative Oncology Group performance status score of 0 or 1.

The median alpha-fetoprotein level was 37.1 ng/mL. The median des-gamma-carboxyprothrombin level was 1780.0 mAU/mL. An infiltrative tumor pattern was identified in 20 (19.6%) patients. The median diameter of the largest measurable lesion was 8.3 cm. Thirty-six (35.3%) patients had multiple tumors, of whom 26 (25.5%) had more than three tumors. Seventy-four (72.5%) patients had uni-lobar tumors, whereas 28 (27.5%) had bi-lobar tumors. Tumor invasion to peripheral branches of the portal vein was identified in 19 (18.6%) patients, of whom eight (7.8%) had portal vein tumor invasion to the first-order branch. Hepatic vein invasion was identified in five (4.9%) patients.

### 3.2. Treatment Outcomes after TARE

**Figure 2:**
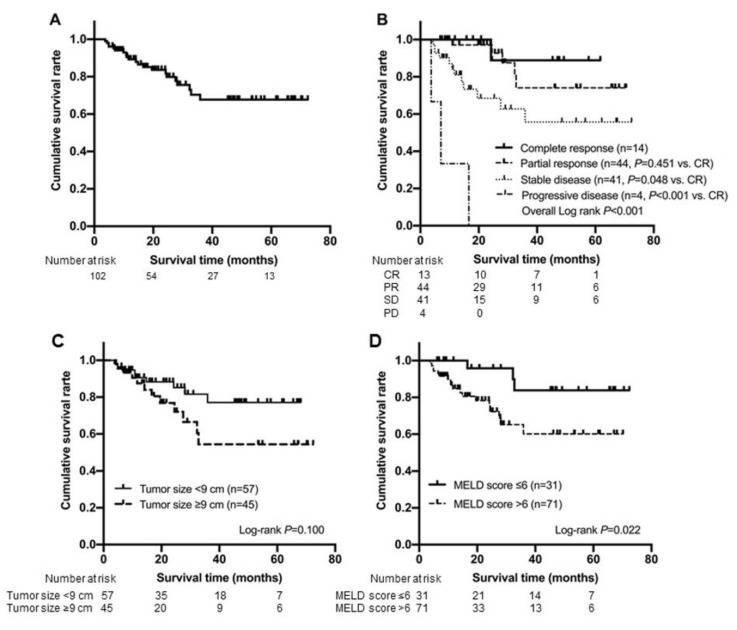
Kaplan-Meier analysis for overall survival of the entire study population (*n* = 102) (**A**) and subgroups according to the best response (**B**). Kaplan-Meier curves for overall survival according to tumor size (**C**) and MELD score (**D**), which were the identified predictors for complete response achievement. mRECIST, modified Response Evaluation Criteria in Solid Tumors; CR, complete response; PR, partial response; SD, stable disease; PD, progressive disease; MELD, model for end-stage liver disease.

During the follow-up period (mean 27.1, median 20.7 months), 21 patients (20.6%) died. The cumulative survival rate after TARE was 96.1% at 6 months, 89.3% at 12 months, and 81.7% at 24 months, respectively. (Figure 2A). As the best response, CR according to mRECIST was achieved in 14 (13.7%) patients, whereas PR, SD, and PD were achieved in 44 (43.1%), 41 (40.2%), and three (2.9%) patients, respectively.

The cumulative survival rate of patients who achieved CR was statistically similar to that of patients who achieved PR (*p* = 0.451 by log-rank test), whereas it was significantly higher than that of patients who achieved SD (*p* = 0.048 by log-rank test) and PD (*p* < 0.001 by log-rank test) (Figure 2B). The survival probabilities of the patients who achieved CR after TARE were 100%, 100%, and 88.9% at 6, 12, and 24 months, respectively. Those of the patients who achieved PR after TARE were 100%, 97.1%, and 92.7% at 6, 12, and 24 months, respectively. Those of the patients who achieved SD after TARE were 90.2%, 77.2%, and 62.7% at 6, 12, and 24 months, respectively. Those of the patients who achieved PD after TARE were 66.7%, 33.3% and 0% at 6, 12, and 24 months, respectively.

During the follow-up period, among 14 patients who achieved CR, CR was maintained in five patients without further treatments, whereas four patients underwent subsequent curative therapy including liver resection (*n* = 3) and liver transplantation (*n* = 1). Four patients underwent subsequent TACE at 7 or 8 months due to local recurrence, and one patient was subsequently treated with intra-arterial infusional chemotherapy owing to early recurrence at 4 months. 

### 3.3. Comparison between Patients with and without CR

Baseline characteristics were compared between patients who achieved CR (*n* = 14, 13.7%) and those who did not (*n* = 88, 86.3%) (Table 2). Patients with CR had favorable baseline laboratory characteristics, such as lower total bilirubin (median 0.6 vs. 0.8 mg/dL), higher serum albumin level (median 4.1 vs. 3.9 g/dL), lower model for end-stage liver disease (MELD) score (median 6.0 vs. 7.0), and lower albumin-bilirubin (ALBI) gradient score (median −2.8 vs. −2.5) compared to patients who did not achieve CR (all *p* < 0.05). Moreover, patients who achieved CR had smaller maximum tumor diameter (median 6.3 vs. 9.0 cm, *p* = 0.012). Other tumor characteristics were statistically similar between the two groups (all *p* > 0.05).

### 3.4. Predictors of CR

In univariate analysis, a higher serum albumin level (>4.1 g/dL), lower aspartate aminotransferase level (<40 or <32 IU/L), lower alkaline phosphatase level, lower MELD score, lower ALBI score, and smaller maximum tumor diameter (<9 cm) significantly predicted an increased probability of CR after TARE (all *p* < 0.05) (Appendix A, Table A1).

Subsequent multivariate analysis based on significant variables in the univariate analysis revealed that lower MELD score (HR = 0.436, 95% CI, 0.224–0.849; *p* = 0.015) and maximum tumor diameter < 9 cm (HR = 11.180, 95% CI, 1.458–85.731; *p* = 0.020) independently predicted an increased probability of CR after TARE (Table 3). 

Cumulative survival rate of patients with maximum tumor diameter < 9 cm and those with MELD score ≤ 6 was significantly longer than those of their counterparts (*p* = 0.100 and *p* = 0.022 by log-rank test, respectively). The survival probabilities of patients with maximum tumor diameter < 9 cm vs. ≥ 9 after TARE were 96.5%, 90.6%, and 88.3% vs. 95.6%, 87.5%, and 76.8% at 6, 12, and 24 months, respectively. In addition, the survival probabilities of the patients with MELD score ≤ 6 vs. > 6 after TARE were 100%, 100%, and 95.8% vs. 94.4%, 84.7%, and 78.2% at 6, 12, and 24 months, respectively (Figure 2C,D).

### 3.5. Pathological Correlation with Radiological CR

Pathological investigation was done in four patients in the CR group (Table 4). Total necrosis was observed in two patients (one with a single tumor and the other with two tumors), and near-total necrosis was noted in two patients (95% necrosis in the larger tumor and 20% necrosis in the smaller tumor in one patient with two tumors, and 95% necrosis in the other patient with a single tumor).

## 4. Discussion

It is important to determine whether the radiological response after locoregional treatment (LRT) for HCC accurately reflects not only the shrinkage in tumor size but also the actual tumor viability. Although the European Association for the Study of the Liver (EASL) criteria and mRECIST are known to be suitable for assessing response and reflecting survival after TACE for HCC [19,20,21,22,23], there is still controversy about which criteria correctly reflect survival after TARE, owing to some confounders including delayed necrosis, peritumoral edema, rim enhancement with granulation tissue, and hemorrhages in the necrotic tumor after TARE [15,27]. Several previous studies have argued that the existing arterial enhancement criteria were not enough to evaluate the long-term prognosis after TARE, and have suggested new response criteria using tumor density or response based on positron emission tomography-CT or MRI to reflect the prognosis after TARE more accurately [28,29,30]. Nevertheless, recent studies have confirmed that CR achievement based on arterial enhancement criteria well reflects the actual complete pathological necrosis (CPN) after LRT, including TARE [29,31].

Therefore, we attempted to identify the independent predictors of mRECIST-based CR in patients with intrahepatic HCC who were treated with TARE.

To our knowledge, this is the first study to identify independent predictors of radiological CR achievement after TARE for intrahepatic HCC. In the present study, 14 (13.7%) patients achieved CR as the best response, in comparison to the previously reported rate (6–32%) of CR achievement according to the enhancement criteria after TARE [11,12,13]. We also found that smaller maximal tumor size (<9 cm) and lower MELD score independently predicted CR according to mRECIST, as the best response for intrahepatic HCC. The OS of each response group was significantly different (57.5 months in the CR group vs. 48.0 months in the SD group vs. 59.2 months in the PR group vs. 9.1 months in the PD group; overall *p* < 0.001, log-rank test). Four of 14 patients in the CR group underwent liver resection or liver transplantation, and showed near-total necrosis of the treated lesion, which was appropriately correlated with the achievement of CR according to mRECIST. Recently, Labgaa et al. reported that 32 of 349 patients (9%) treated with TARE for unresectable HCC could receive curative treatments (22 patients with liver transplantation and 10 with resection); and among them, 18 (56%) patients showed CR according to mRECIST [32]. The study indicated that it might be clinically important to predict CR after TARE to guide the future treatment direction toward curative treatments such as liver transplantation and resection, confirming the therapeutic role of TARE as a bridging or down-staging therapy.

Similar to our study, previous studies have also demonstrated that a small tumor size is a good predictor of tumor response and survival after LRTs [33,34,35] and is also related to CPN [29]. Recently, Riaz et al. reported that CPN was frequently observed in the smaller HCC group treated with TARE (89% of lesions with pretreatment size < 3 cm), and all 12 cases with CR according to the EASL criteria showed perfect CPN [31]. These findings support our results indicating that a small tumor size is an independent predictor of achieving CR according to mRECIST, and that pathological necrosis was well correlated with the radiological response, despite the small number of cases with surgical pathology confirmation. Interestingly, our study showed that relatively large tumors up to 9 cm in diameter could be appropriately treated with TARE, resulting in near-total pathological necrosis, indicating that patients with unresectable intrahepatic HCC might benefit from TARE, if well selected, despite having a large tumor.

In addition, we found that a lower MELD score is another favorable predictor for achieving CR after TARE. Since several studies have revealed that the MELD score might be useful as a general prognostic indicator after TARE for HCC [28,36], our results might indicate that MELD score can be used to predict the long-term outcome and probability of CR achievement. However, the association between MELD score and radiological response after LRT is barely reported in the literature, and further validation studies are required to confirm the exact reason for this association between MELD score and the probability of CR achievement. Additionally, the cutoff for MELD score to significantly discriminate different OS values was only 6. However, our results should be interpreted with caution, as the MELD score of our study population was dichotomized for easier understanding, and our proposed cutoff MELD score of 6 points might have been biased due to the skewed distribution of liver function in our study population. Instead of proposing any specific cutoff value for MELD score, our results might provide the rationale for selecting optimal candidates who may benefit from TARE based on the baseline liver function.

This study had several limitations. First, since it was a retrospective study from a single Korean center with associated selection bias, data might not be representative of real-world situations. In fact, the OS of our study population (mean 55.5 months) seems relatively longer than that reported in other studies on TARE (mean 36 months or median 10–17 months) [11,12,13,37]. Second, although we first tried to identify the potential predictors of CR in univariate analysis and then used subsequent multivariate analysis to identify independent predictors, the over-fitting in multivariate analysis using six variables, compared to the extremely small number of events (only 14 events for CR), might have obviously limited the strength of evidence in our study. Third, as most patients in our study had Child-Pugh class A class liver function (*n* = 96, 94.1%) and hepatitis B-viral infection (*n* = 66 of 71 [92.9%] with viral etiology), the influence of decreased liver function of Child-Pugh B class and hepatitis C viral infection could not be assessed, which should be further investigated in larger sample studies in the future. Fourth, we could not fully assess the association between radiological and pathological responses due to the limited number of surgical cases. Further studies with patients who underwent surgical resection or liver transplantation after TARE can resolve this issue.

## 5. Conclusions

In conclusion, lower MELD score and smaller maximal tumor size independently predicted an increased probability of achieving CR after TARE in patients with unresectable intrahepatic HCC. Our results might help physicians in selecting the optimal candidates who may benefit the most from TARE based on the identified predictors of CR.

## Figures and Tables

**Table 1:** Baseline characteristics of the study population (*n* = 102).

Variables	Values
Age	64.3 (53.6–72.8)
Male sex	83 (81.4)
Body mass index, kg/m^2^	23.9 (21.3–25.8)
Diabetes mellitus	44 (43.1)
Hypertension	59 (57.8)
Liver cirrhosis	61 (55.5)
Viral etiology	71 (69.6)
Heavy alcoholics	6 (5.9)
Platelet count, ×10^9^/L	198.5 (121.3–261.5)
Total bilirubin, mg/dL	0.6 (0.5–0.9)
Serum albumin, g/dL	3.9 (3.5–4.1)
Aspartate aminotransferase, IU/L	40.0 (28.8–64.3)
Alanine aminotransferase, IU/L	28.5 (18.0–45.0)
Alkaline phosphatase, IU/L	95.5 (76.0–138.5)
Prothrombin time, INR	1.0 (0.96–1.10)
AFP, ng/mL	37.1 (5.0–2590.1)
DCP, mAU/mL	1780.0 (135.5–8119.5)
MELD score	7.6 (6–8)
ALBI grade	
Grade 1/2/3	52 (51.0)/47 (46.1)/3 (2.9)
Tumor pattern	
Nodular/infiltrative	82 (80.4)/20 (19.6)
Maximum tumor diameter, cm	8.3 (6.0–10.5)
Multiple tumors	36 (35.3)
Tumor burden >50%	10 (9.8)
Tumor distribution	
Uni-lobar/bi-lobar	74 (72.5)/28 (27.5)
Portal vein thrombosis	19 (18.6)
First-/second-/segmental-order branch	8/6/5
Hepatic vein invasion	5 (4.9)

Variables are expressed as median (interquartile range) or *n* (%). INR, international normalized ratio; AFP, alpha-fetoprotein; DCP, des-gamma-carboxyprothrombin; MELD, model for end-stage liver disease; ALBI, albumin-bilirubin.

**Table 2:** Comparison between patients who achieved complete response (CR) and those who did not.

Variables	CR Group (*n* = 14, 13.7%)	Non-CR Group (*n* = 88, 86.3%)	*p*-Value
Age	62.0 (51.1–75.2)	64.6 (53.8–72.7)	0.993
Male sex	14 (100.0)	69 (78.4)	0.054
Body mass index, kg/m^2^	24.4 (22.0–26.9)	23.7 (21.3–25.6)	0.539
Diabetes mellitus	9 (64.3)	35 (39.8)	0.085
Hypertension	8 (57.1)	51 (58.0)	0.954
Liver cirrhosis	11 (78.6)	50 (56.8)	0.123
Viral etiology	11 (78.6)	60 (68.2)	0.432
Heavy alcoholics	2 (14.3)	4 (4.5)	0.150
Platelet count, ×10^9^/L	160.0 (96.3–202.5)	204.0 (140.0–272.8)	0.056
Bilirubin, mg/dL	0.6 (0.5–0.7)	0.8 (0.5–1.0)	0.006
Albumin, g/dL	4.1 (3.8–4.3)	3.9 (3.5–4.1)	0.031
Aspartate aminotransferase, IU/L	30.0 (23.5–35.0)	43.0 (30.0–66.0)	<0.001
Alanine aminotransferase, IU/L	24.0 (22.0–35.8)	31.0 (18.0–46.5)	0.057
Alkaline phosphatase, IU/L	79.0 (64.8–97.5)	103.0 (77.0–156.0)	<0.001
Prothrombin time, INR	1.0 (1.0–1.1)	1.0 (1.0–1.1)	0.702
AFP, ng/mL	12.7 (3.1–284.9)	39.9 (5.2–3078.0)	0.001
DCP, mAU/mL	575.5 (33.0–2000.8)	2772.0 (5.2–3078.0)	0.003
MELD score	6.0 (6.0–7.0)	7.0 (6.0–8.0)	<0.001
ALBI score	−2.8 (−2.9 to −2.5)	−2.5 (−2.8 to −2.2)	0.024
Grade 1/2/3	10 (71.4)/4 (28.6)/0 (0)	42 (47.7)/43 (48.9)/3(0.03)	0.099
Infiltrative tumor pattern	4 (28.6)	16 (18.2)	0.363
Maximum tumor diameter, cm	6.3 (5.2–8.3)	9.0 (6.4–11.4)	0.012
Multiple tumors	4 (28.6)	32 (36.4)	0.571
Tumor burden >50%	0 (0)	10 (11.4)	-
Bi-lobar tumor distribution	1 (7.1)	27 (30.7)	0.067
Portal vein thrombosis	1 (7.1)	18 (20.5)	0.458
First-/second-/segmental-order branch	0/0/1	8/6/4	-
Hepatic vein invasion	0 (0.0)	5 (5.7)	-

Variables are expressed as median (interquartile range) or *n* (%). CR, complete response; INR, international normalized ratio; AFP, alpha-fetoprotein; DCP, des-gamma-carboxyprothrombin; MELD, model for end-stage liver disease; ALBI, albumin-bilirubin.

**Table 3:** Results of multivariate Cox regression analysis to identify predictors of complete response.

Variables	Univariate	Multivariate Analysis	
	*p*-Value	*p*-Value	Hazards Ratio (95% CI)
Albumin, g/dL	0.036	0.588	3.685 (0.033–415.239)
ALBI score	0.028	0.859	1.548 (0.013–189.867)
AST <32 IU/L	0.016	0.361	1.846 (0.495–6.889)
ALP <90 IU/L	0.042	0.573	1.526 (0.351–6.629)
MELD score	0.018	0.015	0.436 (0.224–0.849)
Maximum tumor diameter 9 cm	0.015	0.020	11.180 (1.458–85.731)

ALBI, albumin-bilirubin; AST, aspartate aminotransferase; ALP, alkaline phosphatase; MELD, model for end-stage liver disease; CI, confidence interval.

**Table 4:** Pathological information of patients who achieved complete response.

Patient	Age at TARE, Years	Sex	MELD Score	Tumor Number	Maximum Tumor Diameter, cm	Duration between TARE and Operation, Month	Type of Curative Resection	Pathological Findings
A	58	Male	6	1	6.0	17	Resection	Total necrosis
B	58	Male	6	3	8.8	7	Resection	Total necrosis
C	57	Male	7	2	8.2	14	Liver transplantation	95% necrosis of the largest mass, 20% necrosis of the small mass
D	54	Male	7	1	8.5	3	S5, 6 segmentectomy as a scheduled bridging operation	95% necrosis

TARE, trans-arterial radioembolization; MELD, model for end-stage liver disease.

## Data Availability

Not applicable.

## Appendix A

**Table A1:** Univariate Cox-regression analysis to predict CR.

Variable	Univariate Analysis	
	*p* Value	Hazards Ratio (95% CI)
Age > 65 years	0.735	0.821(0.263–2.564)
Male gender	0.998	-
Body mass index, kg/m^2^	0.535	1.052 (0.896–1.235)
Diabetes mellitus	0.094	2.726 (0.843–8.814)
Hypertension	0.954	0.967 (0.309–3.025)
Liver cirrhosis	0.135	2.787 (0.726–10.690)
Viral etiology	0.437	1.711 (0.442–6.621)
Heavy alcoholics	0.173	3.500 (0.577–21.215)
Platelet count, ×10^9^/L	0.061	0.993 (0.986–1.000)
Total bilirubin, mg/dL	0.124	0.213 (0.030–1.530)
Serum albumin, g/dL	0.036	5.864 (1.121–30.672)
>4.1 g/dL	0.029	3.368 (1.129–10.052)
Aspartate aminotransferase, IU/L	0.014	0.937 (0.889–0.987)
<40 IU/L	0.021	11.016 (1.441–84.242)
<32 IU/L	0.016	4.292 (1.312–14.042)
Alanine aminotransferase, IU/L	0.220	0.978 (0.945–1.013)
Alkaline phosphatase, IU/L	0.037	0.979 (0.959–0.999)
Prothrombin time, INR	0.699	0.340 (0.001–80.476)
AFP, ng/mL	0.381	1.000 (1.000–1.000)
DCP, mAU/mL	0.226	1.000 (1.000–1.000)
MELD score	0.018	0.440 (0.223–0.871)
ALBI score	0.025	0.169 (0.035–0.801)
ALBI grade 2 (vs. grade 1)	0.149	0.426 (0.133–1.357)
Infiltrative tumor pattern	0.368	1.800 (0.501–6.473)
Maximum tumor diameter, cm	0.016	0.746 (0.588–0.946)
<9 cm	0.015	13.0 (1.630–103.687)
Multiple tumors	0.572	1.429 (0.414–4.928)
Bi-lobar tumor distribution	0.100	0.174 (0.022–1.396)
Portal vein thrombosis	0.260	0.299 (0.037–2.440)

CR, complete response; INR, international normalized ratio; AFP, alpha-fetoprotein; DCP, des-gamma-carboxyprothrombin; MELD, model for end-stage liver disease; ALBI, Albumin-Bilirubin; CI, confidence interval.
